# Identification and Thermal Stability Modification of a Zearalenone Lactone Hydrolase ZHD30L

**DOI:** 10.3390/toxins18080324

**Published:** 2026-07-26

**Authors:** Xingsai Liu, Fengguang Zhao, Kashif Iqbal Sahibzada, Yaping Zhang, Shan Wei, Pingping Tian, Yingying Wang, Yuansen Hu, Yangyong Lv

**Affiliations:** 1 Food Laboratory of Zhongyuan, Luohe 462300, China; lxs2810@stu.haut.edu.cn; 2 College of Biological Engineering, Henan University of Technology, Zhengzhou 450001, China; 3 Department of Health Professional Technologies, Faculty of Allied Health Sciences, The University of Lahore, Lahore 54570, Pakistan; 4 College of Food and Bioengineering, Henan University of Science and Technology, Luoyang 471023, China

**Keywords:** zearalenone, thermostability, semi-rational design, molecular dynamics simulation

## Abstract

In this study, a previously uncharacterized zearalenone lactone hydrolase, ZHD30L, from *Knufia peltigerae* was identified and characterized, and semi-rational design was employed to enhance its thermostability. The results indicate that the optimal reaction conditions for this enzyme are 40 °C and pH 9.0, under which it catalyzes the ring-opening of the ZEN lactone to generate the non-toxic product HZEN. The mutant with five combined sites Mut5, (E11T-A127V-I180K-H235P-V156I), was obtained through semi-rational design combined with multiple rounds of iterative superposition and negative elimination strategies. Thermodynamic characterization revealed that, compared to the wild type, the t_1/2_ of Mut5 at 48 °C was significantly extended from 1.67 min to 796.8 min, and the *T*_m_ value increased from 39.56 °C to 50.04 °C. The markedly prolonged thermal half-life and increased apparent melting temperature may improve the operational robustness of Mut5 during moderate-temperature feed and food processing or post-processing applications, potentially reducing activity loss and the need for repeated enzyme additions. Concurrently, the *k_cat_*/*K_M_* improved from 19.08 μM^−1^·s^−1^ (95% Cl = 12.80 to 25.36 μM^−1^·s^−1^) to 33.29 μM^−1^·s^−1^ (95% Cl = 23.20 to 43.38 μM^−1^·s^−1^). Under optimal reaction conditions, WT and Mut5 degraded 76.51% ± 1.65% and 99.13% ± 0.71% of ZEN (10 μg/mL), respectively, within 3 min at equal enzyme concentrations. Molecular dynamics simulations indicate that the H235P and E11T mutations reduce local conformational entropy, the A127V and V156I mutations optimize internal hydrophobic stacking, and the I180K mutation reshapes the local charge microenvironment. Furthermore, Mut5 exhibited an overall RMSD at high temperatures that was 0.026 Å lower than that of the wild type, and a global free energy minimum that was 0.36 kcal/mol lower. These computational results offer possible structural interpretations, providing a theoretical reference for developing highly efficient enzyme preparations for the feed and food industries.

## 1. Introduction

Zearalenone (ZEN) is a non-steroidal estrogenic mycotoxin produced by *Fusarium* spp. Up to 60–80% of crops worldwide are at risk of latent mycotoxin contamination, with the detection rate of ZEN showing a significant upward trend [1]. ZEN widely contaminates cereal crops and poses a severe threat to the reproductive health of livestock and poultry [2]. As a resorcylic acid lactone compound, the spatial conformation of its 14-membered lactone ring is highly analogous to that of 17 β-estradiol. The structural similarity enables ZEN to competitively bind to estrogen receptors in animals, thereby inducing severe reproductive disorders, endocrine disruption, and genotoxicity [3]. Moreover, ZEN causes severe systemic oxidative stress and immunosuppression, with subclinical damage to multiple organs [4]. Physiochemical detoxification is one of the available methods for detoxification but has limitations such as poor substrate specificity, high risk of secondary pollution, and severe destruction of nutritional components [5,6]. Consequently, using lactonases (ZHD) to cleave the lactone ring structure of ZEN, followed by its conversion to the non-toxic decarboxylated product DHZEN, is widely regarded as the most promising green detoxification strategy for practical applications [7,8]. However, naturally discovered ZHDs universally face the bottleneck of insufficient thermostability in industrial applications, which limits their practical efficacy in high-temperature processing environments, thus urgently necessitating molecular modification through protein engineering approaches [9].

Representative microbial ZEN lactone hydrolases include ZHD101 from *Clonostachys rosea* [7], ZENG from *Gliocladium roseum* [10], ZHRnZ from *Rosellinia necatrix* [11], and ZHD11A from *Phialophora macrospora* [12]. However, the thermodynamic stability of these natural enzymes does not meet the demands of practical applications. The optimal temperatures for most reported ZHD family enzymes range from 30 to 45 °C [13]. Several reported natural ZEN lactone hydrolases exhibit limited thermal tolerance and rapidly lose activity when incubated at elevated temperatures [14]. For example, ZHRnZ is rapidly inactivated above 40 °C [11], whereas CbZHD loses detectable activity after incubation at 45 °C for 2 min [15]. ZHD607 retained 56% of its initial activity after 10 min at 40 °C and 60% of its activity after 30 min at 35 °C [16]. This inherent deficiency in natural thermostability severely restricts the large-scale industrial application of ZEN detoxifying enzymes [17]. Additionally, the industrial translation of enzymatic ZEN detoxification has recently started to progress beyond laboratory-scale studies. A ZenA-based zearalenone hydrolase from Biomin GmbH has been evaluated by the European Food Safety Authority (EFSA) for use as a feed additive and has been incorporated into commercial mycotoxin-management products in selected markets [18]. These products are primarily intended to transform ZEN in the gastrointestinal tract after ingestion [19]. However, enzyme stability during manufacturing, formulation, storage, and exposure to complex feed matrices remains an important consideration for broader industrial implementation. Consequently, the development of ZEN hydrolases with improved thermal and operational stability continues to be of considerable practical interest.

Protein-engineering strategies have been explored in several studies involving ZHD101-like zearalenone lactone hydrolases (ZHDs) to improve their thermal stability. General computational tools such as the PROSS algorithm, which combines phylogenetic analysis with Rosetta atomic design [20,21,22], and FoldX, which estimates the free energy of fold change (ΔΔG) [23], offer approaches for prioritizing potentially stabilizing substitutions, yet only a limited number of studies have experimentally applied such strategies to ZHD-family enzymes. For example, Yu et al. combined FireProt and PROSS-guided design, ΔΔG-based screening, and structural filtering resulting in the successful identification of the A59P variant with enhanced thermal stability [24]. Guan et al. identified the S220R and S220W mutants, exhibiting the best thermal stability, through structural alignment and ΔΔG calculations [25].

In this study, we aimed to identify and biochemically characterize the previously uncharacterized ZEN lactone hydrolase ZHD30L from *Knufia peltigerae*; improve its thermostability through PROSS and Rosetta-assisted semi-rational design followed by iterative experimental screening; verify whether the resulting mutants could achieve enhanced stability without compromising catalytic performance; and use molecular docking and molecular dynamics simulations to explore the basis of the stability improvement. We hypothesized that amino acid substitutions in noncatalytic regions could improve global structural stability while preserving the local conformational properties required for substrate turnover. A five-mutation variant, Mut5 (E11T-A127V-I180K-H235P-V156I), was successfully constructed through iterative multi-site screening, showing both improved thermostability and stable catalytic activity. Molecular dynamics simulations and structural analyses provided possible explanations for the improved thermal stability and catalytic performance of Mut5.

## 2. Results

### 2.1. Sequence Characteristics of the ZEN Hydrolase ZHD30L

**Figure 1:**
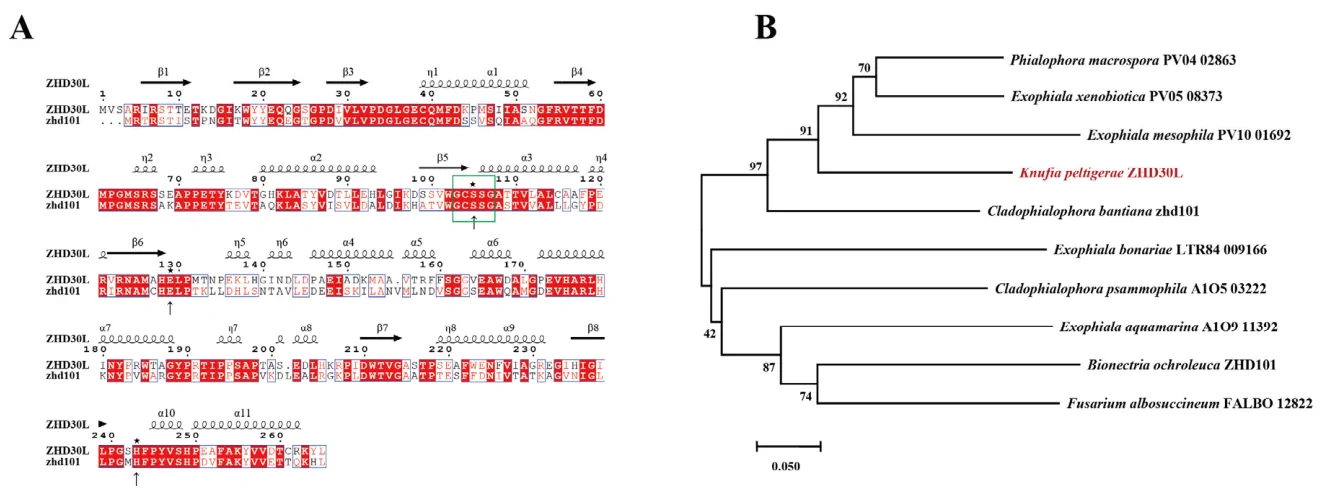
Sequence characteristics and evolutionary analysis of ZHD30L. (**A**) Sequence alignment of ZHD30L with classic lactonases; (**B**) Phylogenetic analysis of ZHD30L. The arrows indicate the catalytic triad residues Ser, Glu, and His, while asterisks denote highly conserved resi-dues.

Through homology-based alignment, a novel lactonase, ZHD30L, derived from *Knufia peltigerae*, was identified. Amino acid sequence alignment analysis showed that ZHD30L shares 71.7% homology with the known template ZHD101 and retains the typical Ser-Glu-His catalytic triad and the G-C-S-S-G conserved motif. Secondary structure annotation results based on ESPript 3.0 sequence analysis predicted that ZHD30L contains 11 α-helices and eight β-strands (Figure 1A), broadly resembling the α/β-hydrolase fold reported for ZHD101. This comparison is prediction-based and does not represent an experimentally solved structure of ZHD30L. Although ZHD30L and ZHD101 were located on separate branches in the phylogenetic analysis (Figure 1B), their high sequence identity, conserved catalytic motif and predicted secondary-structure composition support a broadly conserved ZHD-family α/β-hydrolase fold. Biochemical characterization was therefore used to determine the functional properties of ZHD30L.

### 2.2. Characterization of Enzymatic Properties of the WT

**Figure 2:**
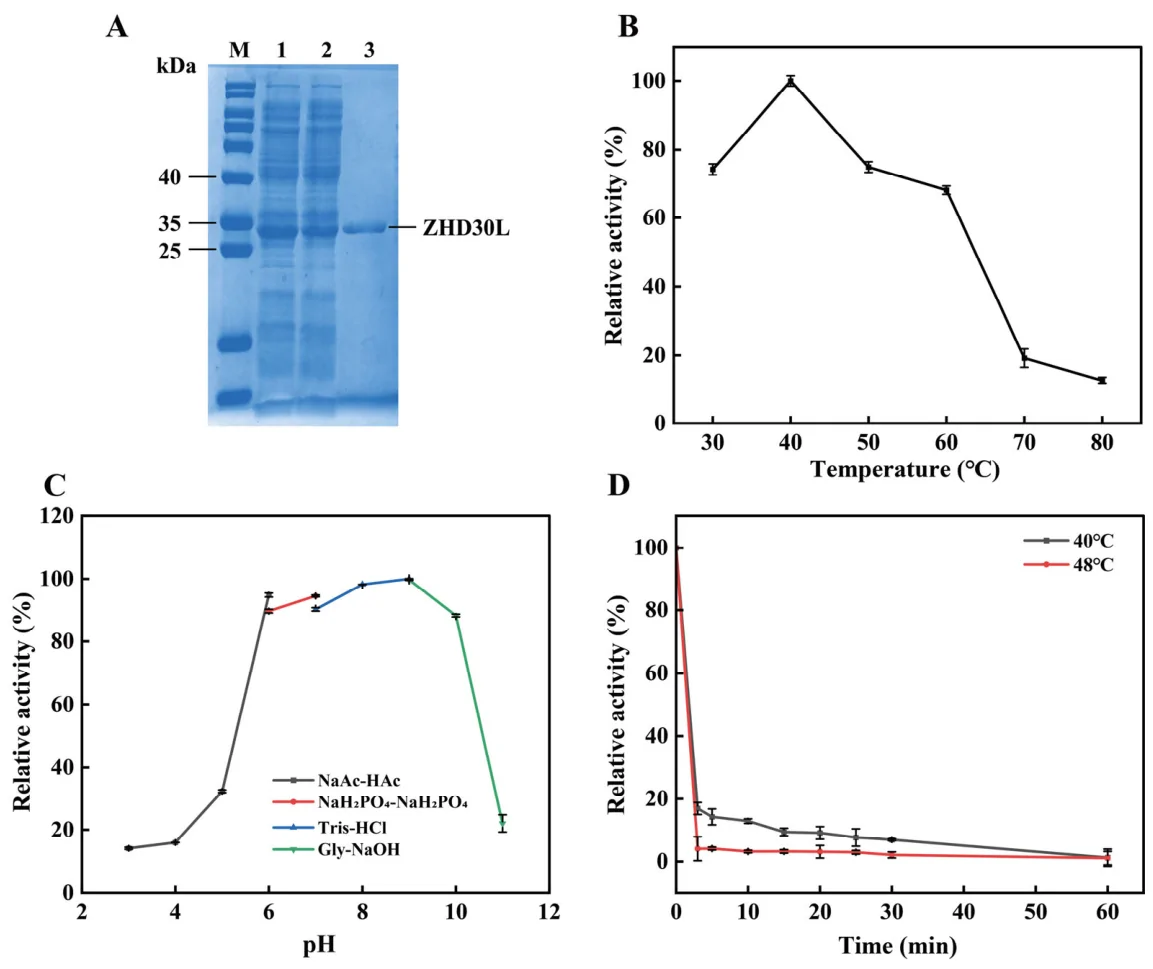
Enzymatic properties of ZHD30L. (**A**) SDS-PAGE analysis of ZHD30L, where M represents a 10–180 kDa protein marker; lane 1: cell lysate supernatant, lane 2: elution buffer, lane 3: 200 mM imidazole elution buffer; (**B**) Optimal temperature; (**C**) Optimal pH; (**D**) Thermal stability.

SDS-PAGE results showed that, compared to the cell lysate supernatant, the target protein band in the 200 mM imidazole elution buffer was clear and distinct, indicating that most non-specific contaminating proteins had been removed (Figure 2A). For optimal-condition and thermostability assays of the purified ZHD30L, ZEN conversion was quantified by HPLC measurement of residual ZEN as described in Section 5.5. Physicochemical characterization revealed that the WT has an optimal temperature of 40 °C and an optimal reaction pH of 9.0 (Figure 2B,C). However, the WT is highly sensitive to high temperatures. After incubation at 40 °C and 48 °C for 3 min, its residual enzyme activity decreased to below 20% and 5%, respectively (Figure 2D). The pH stability results (Figure S1A) indicated that ZHD30L has excellent tolerance to neutral and weakly alkaline environments. After treatment in pH 5.0–10.0 buffers for 12 h, the residual enzyme activity remained above 80%. However, under strongly acidic conditions (pH < 5.0), the loss of enzyme activity was significant. Under the tested conditions, most metal ions had relatively limited effects on ZHD30L activity, whereas Cu^2+^, Ni^2+^, and Zn^2+^ decreased the residual activity. Cu^2+^ and Zn^2+^ produced the strongest inhibition, while Ni^2+^ produced a more moderate inhibitory effect (Figure S1B).

### 2.3. LC-MS/MS Analysis of ZEN Hydrolysis Products Generated by ZHD30L

**Figure 3:**
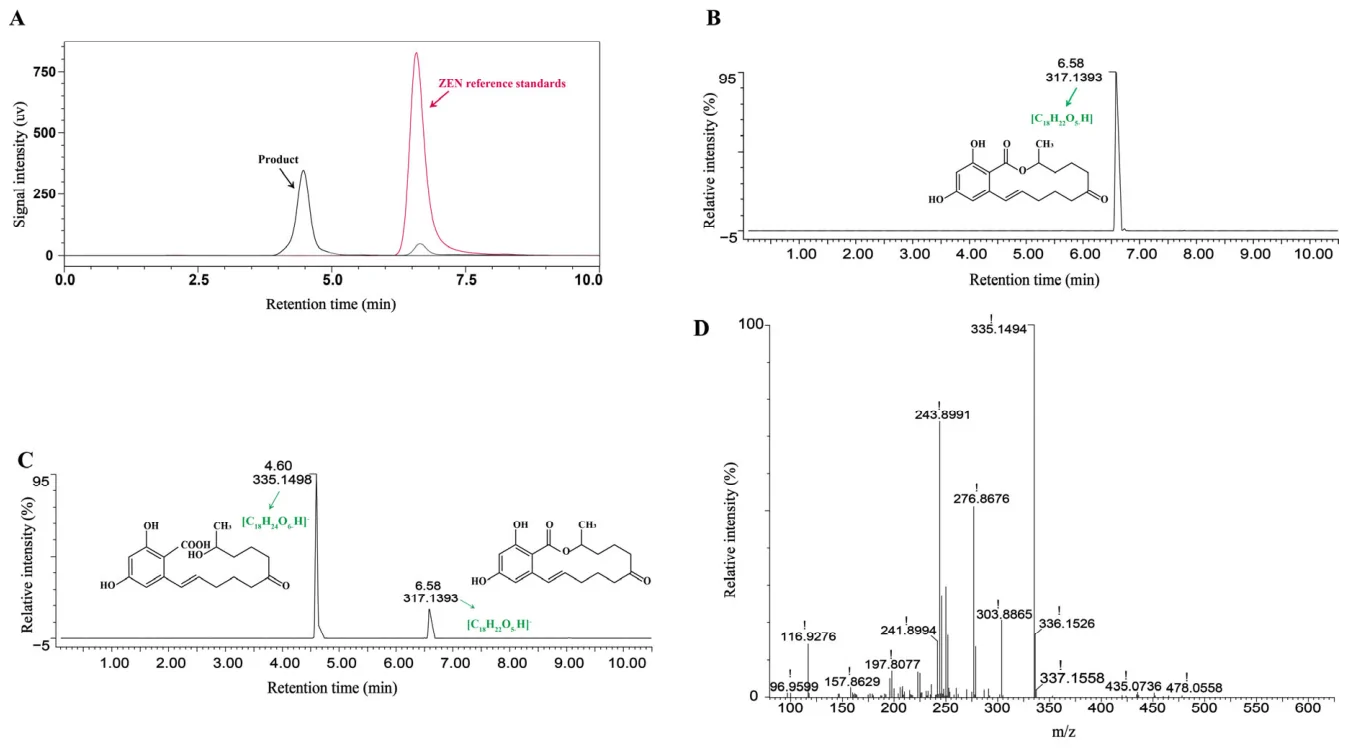
Degradation of ZEN catalyzed by ZHD30L. (**A**) HPLC analysis of ZHD30L degradation of ZEN; (**B**) EIC chromatogram of the control group; (**C**) EIC chromatogram of the sample group; (**D**) Full MS/MS spectrum of the degradation products.

HPLC analysis of ZEN degradation by ZHD30L showed that a peak corresponding to the degradation product appeared at a retention time of approximately 4.5 min (Figure 3A). Mass spectrometry results revealed that ZHD30L specifically hydrolyzes the ester bond of the 14-membered lactone ring of the substrate ZEN (*m*/*z* 317.13) (Figure 3B), converting it into the intermediate HZEN (*m*/*z* 335.1494) with an open side chain (Figure 3C, D).

### 2.4. Mutant Screening and Combinational Evolution Based on the PROSS Algorithm

**Figure 4:**
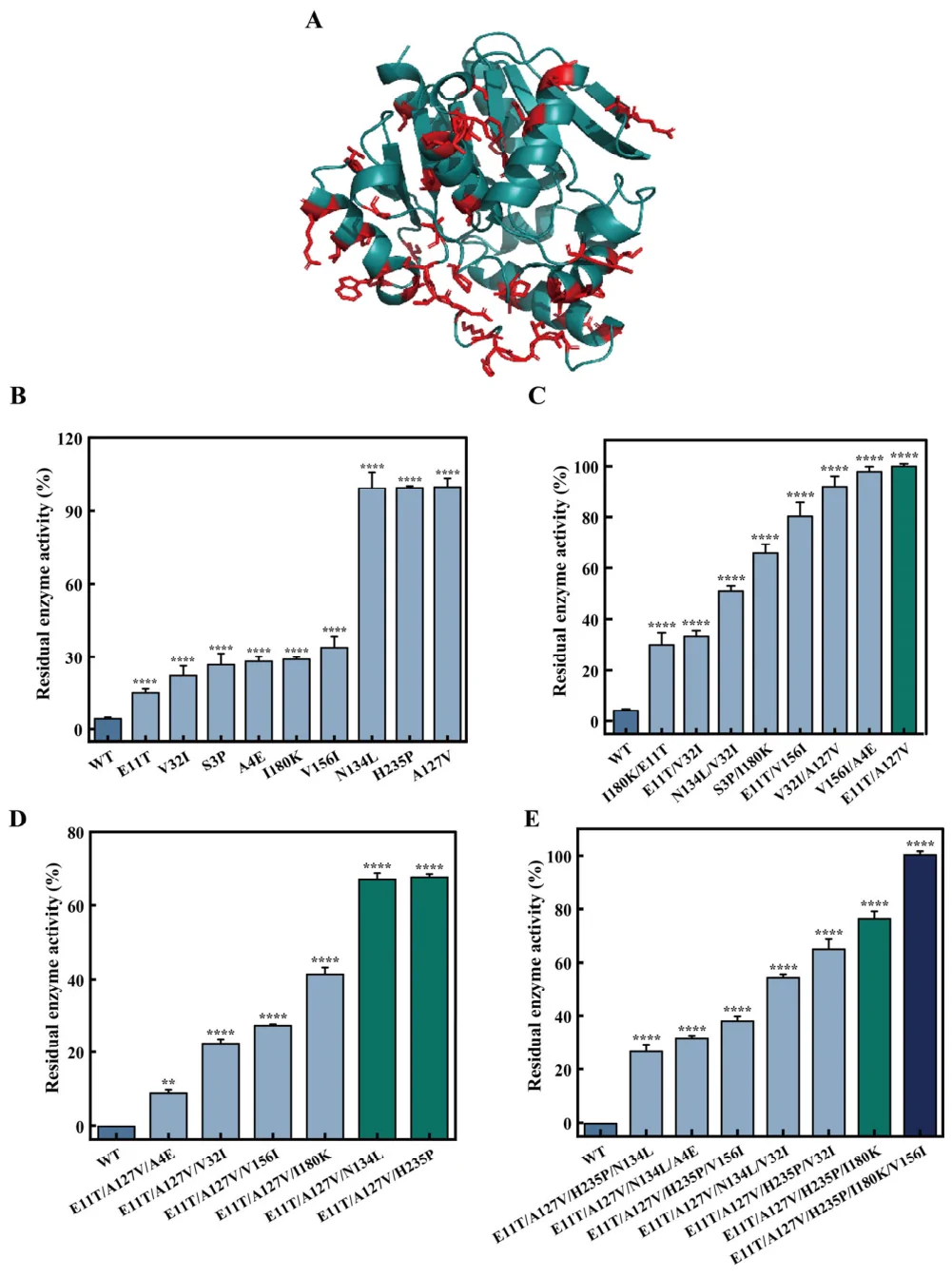
Mutant design and screening based on the PROSS algorithm. (**A**) Distribution of 50 mutation sites on the 3D model; (**B**) Residual enzyme activity of single-point mutants; (**C**) Residual enzyme activity of double-combination mutants; (**D**) Residual enzyme activity of triple-combination mutants; (**E**) Residual enzyme activity of four-and five-combination mutants. **** indicates a significant difference at *p* < 0.0001, ** indicates a significant difference at *p* < 0.01.

Based on the homology model (PDB ID: 5z5j.1.A, crystal structure of a lactonase double mutant from *Rhinocladiella mackenziei*, sequence identity 74.14%), 50 potential thermostable mutation sites were predicted using the PROSS algorithm integrated with ΔΔG calculations (Figure 4A). The results showed that the residual activity of the wild type was only 4.2% at 48 °C for 3 min, whereas 9 single-point mutants demonstrated significantly improved thermal tolerance (Figure 4B). To further investigate the synergistic effects of multiple mutation sites, combination mutagenesis was performed based on these 9 beneficial single-point mutants (Figure 4C,D,E). The combination substitution mutants were selected through iterative experimental screening rather than being solely on the basis of computational single-point prediction. Starting from the beneficial single-point variants, the best-performing mutant in each round (E11T-A127V, E11T-A127V-H235P, E11T-A127V-H235P-I180K) was used as the scaffold for the next round of site stacking. By stepwise stacking of beneficial sites and eliminating those with negative synergistic effects, a five-substitution mutant, Mut5 (E11T-A127V-I180K-H235P-V156I), was successfully obtained (Figure 4E). This mutant maintained nearly 100% residual activity even at 48 °C for 80 min, whereas the addition of other candidate substitutions provided no further benefit or resulted in negative epistatic effects.

### 2.5. Enzymatic Property Analysis of the Combined Mutant Mut5

**Figure 5:**
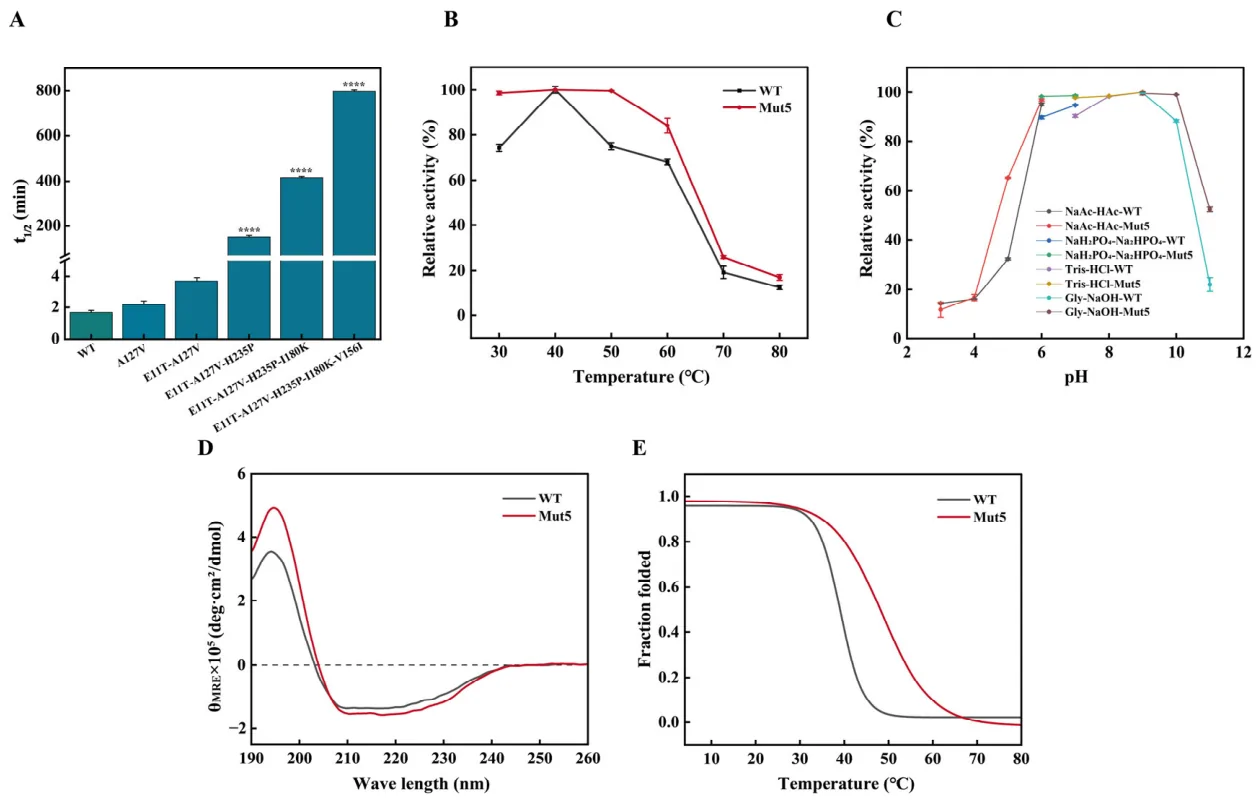
Comparison of enzymatic properties and thermodynamic parameters between WT and Mut5. (**A**) t_1/2_ of the optimal mutants from each round; B. Comparison of optimal temperature between WT and Mut5; (**B**) Comparison of optimal temperature between WT and Mut5; (**C**) Comparison of optimal pH between WT and Mut5; (**D**) CD spectra of WT and Mut5; (**E**) *T*_m_ curves of WT and Mut5. **** indicates a significant difference at *p* < 0.0001.

The results showed that at 48 °C, the t_1/2_ of Mut5 was significantly extended to 796.8 min, an increase of nearly 477-fold compared to WT (1.67 min) (Figure 5A). The optimal temperature and pH of Mut5 were the same as those of WT, at 40 °C and 9.0, respectively; however, the enzyme displayed a broader operational temperature range after mutation (Figure 5B,C). Under the optimal reaction conditions, WT and Mut5 degraded 76.51% ± 1.65% and 99.13% ± 0.71% of ZEN (10 μg/mL), respectively, within 3 min at an equal enzyme concentration. Temperature-dependent CD measurements were performed by monitoring ellipticity at 220 nm during heating from 10 to 80 °C at 1 °C min^−1^. The resulting thermal unfolding curves yielded apparent *T*_m_ values of 39.56 °C for WT and 50.04 °C for Mut5. (Figure 5D,E). In protein engineering, enhancing thermostability often reduces catalytic activity. However, kinetic parameter determination showed that the *K_M_* value of Mut5 for ZEN decreased from 74.77 μM (95% Cl = 57.17 to 92.37 μM) to 64.41 μM (95% Cl = 49.99 to 78.83 μM), and the *k_cat_* value increased from 1427 s^−1^ (95% Cl = 1100.52 to 1753.48 s^−1^) to 2144 s^−1^ (95% Cl = 1705.16 to 2582.84 s^−1^). As a result, the *k_cat_*/*K_M_* increased from 19.08 μM^−1^·s^−1^ (95% Cl = 12.80 to 25.36 μM^−1^·s^−1^) to 33.29 μM^−1^·s^−1^ (95% Cl = 23.20 to 43.38 μM^−1^·s^−1^). This indicates that Mut5 showed a higher apparent catalytic turnover and catalytic efficiency than WT, whereas the overlapping *K_M_* values indicate broadly comparable apparent substrate-binding characteristics under the assay conditions (Table 1).

### 2.6. Local Structure and Electrostatic Potential Analysis of Mutation Sites

**Figure 6:**
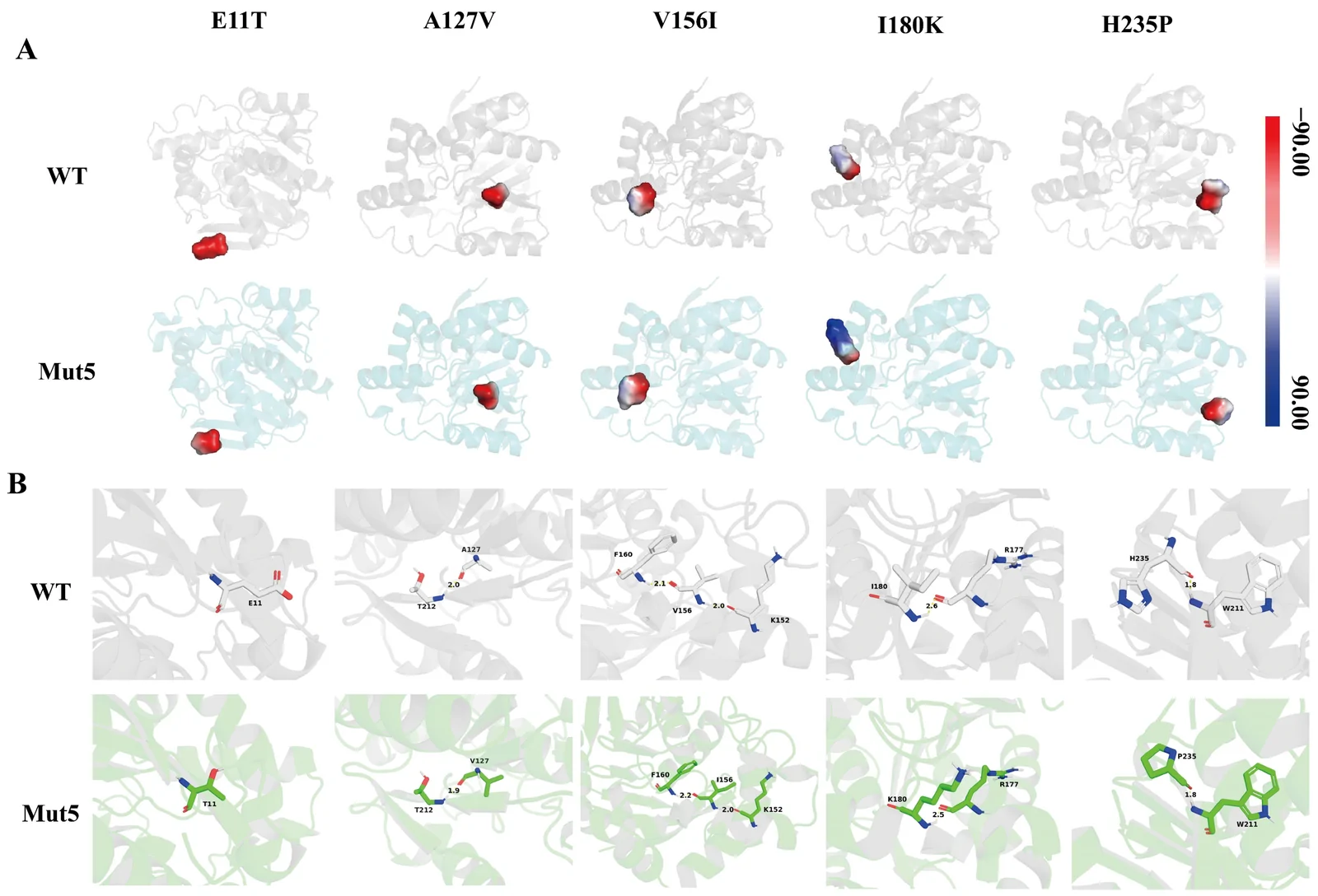
Local structure and electrostatic potential analysis of the five key mutation sites. (**A**) Local structure diagrams; (**B**) ESP maps.

By analyzing the local three-dimensional structures and electrostatic surface potentials (ESP) of the five key mutation sites (E11T, A127V, I180K, H235P, and V156I), the potential mechanisms underlying the dual enhancement of stability and activity in Mut5 were investigated. Local structural alignments showed that the mutations significantly reshaped the non-covalent interaction networks in critical regions (Figure 6A). The proline introduced by the H235P mutation restricted the rotation of main-chain dihedral angles, effectively reducing the flexibility and conformational entropy of the local loop region. The larger hydrophobic side chains introduced by the A127V and V156I mutations filled the internal hydrophobic cavities of the protein, increasing the packing density in the core region. Furthermore, ESP analysis indicated that the I180K and E11T mutations altered the local surface electrostatic distribution, reshaping the polar microenvironment at the protein-solvent interface (Figure 6B). This synergistic optimization of backbone hydrophobic densification and the surface electrostatic network provided a structural foundation for the enhancement of overall thermodynamic stability and the improvement of catalytic pocket interactions.

### 2.7. Molecular Docking and Binding Free Energy Analysis

**Figure 7:**
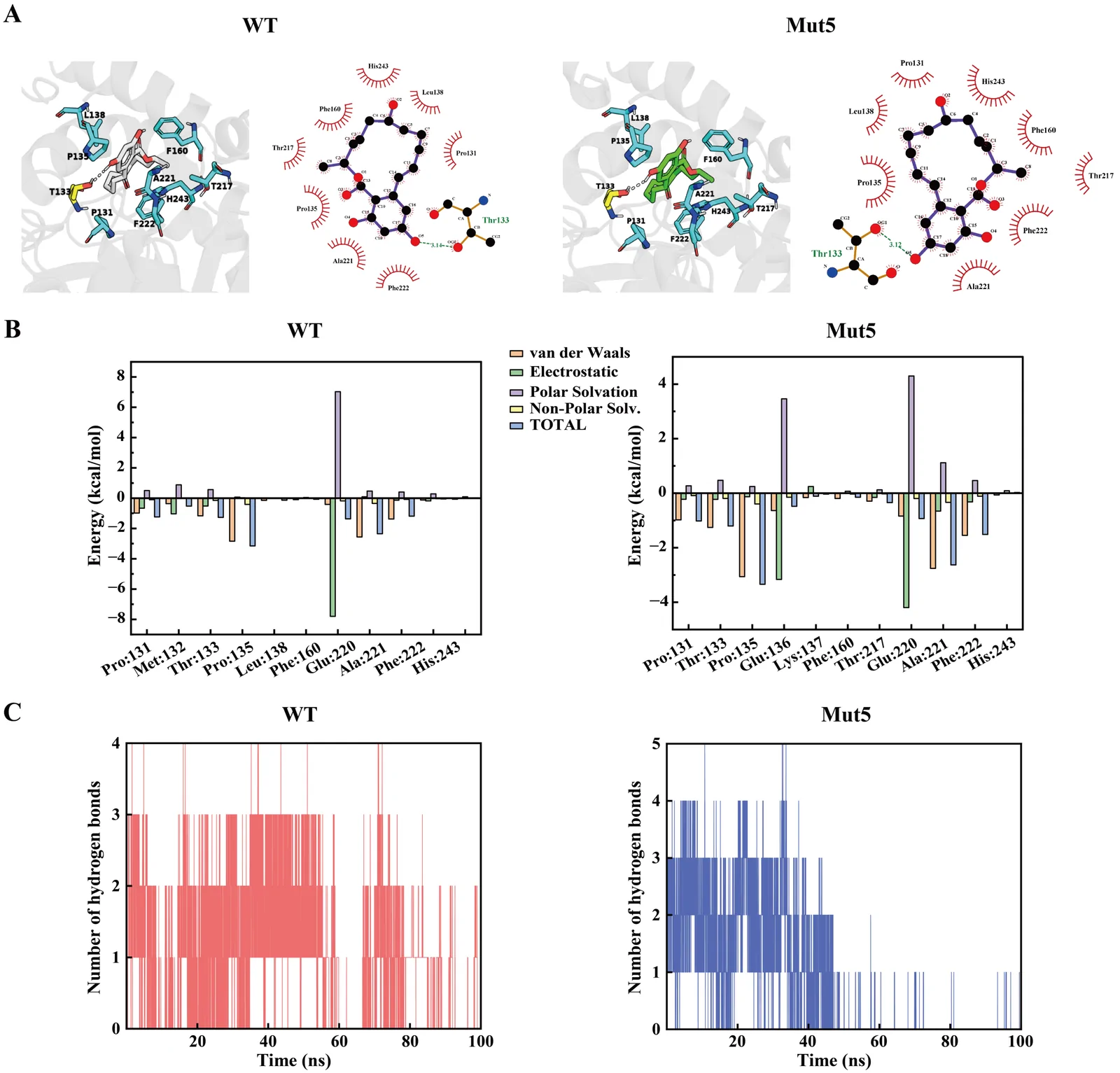
Substrate binding mode and binding free energy analysis. (**A**) Binding cavities and substrate binding modes of WT and Mut5; (**B**) Binding free energy and residue energy contribution plots; (**C**) Number of hydrogen bonds formed between ZEN and protein residues in the WT-ZEN and Mut5-ZEN complexes during the 100 ns molecular-dynamics trajectories.

Molecular docking results indicate that both WT and Mut5 can stably bind to the substrate through the hydrophobic pocket and hydrogen bond network of the active site. Furthermore, the hydrogen bond length between the substrate and the catalytic residue Thr133 was reduced from 3.14 Å in the wild type to 3.12 Å, suggesting that Mut5 exhibits higher substrate binding affinity than the wild type (Figure 7A). To evaluate the binding affinity of the protein–ligand complex and elucidate the energy contributions of key residues, the binding free energy was calculated and decomposed using the MM/GBSA method [26]. Compared with WT, Ala221 and Phe222 showed more favorable per-residue energy contributions in Mut5, with the corresponding values changing from −2.35 and −1.19 kcal/mol to −2.63 and −1.51 kcal/mol, respectively. In addition, residues such as Thr133, Glu220, and Phe222 also contribute to the stabilization of the binding process, further enriching the interaction network at the binding interface (Figure 7B). Although the 100 ns MD time-series analysis showed that the total number of hydrogen bonds maintained within the WT system was slightly higher (Figure 7C), Mut5 compensated for this and achieved comparable affinity through the reinforcement of the aforementioned key local polar interactions and the reshaping of the hydrophobic network.

### 2.8. Structural Stability and Flexibility Analysis

**Figure 8:**
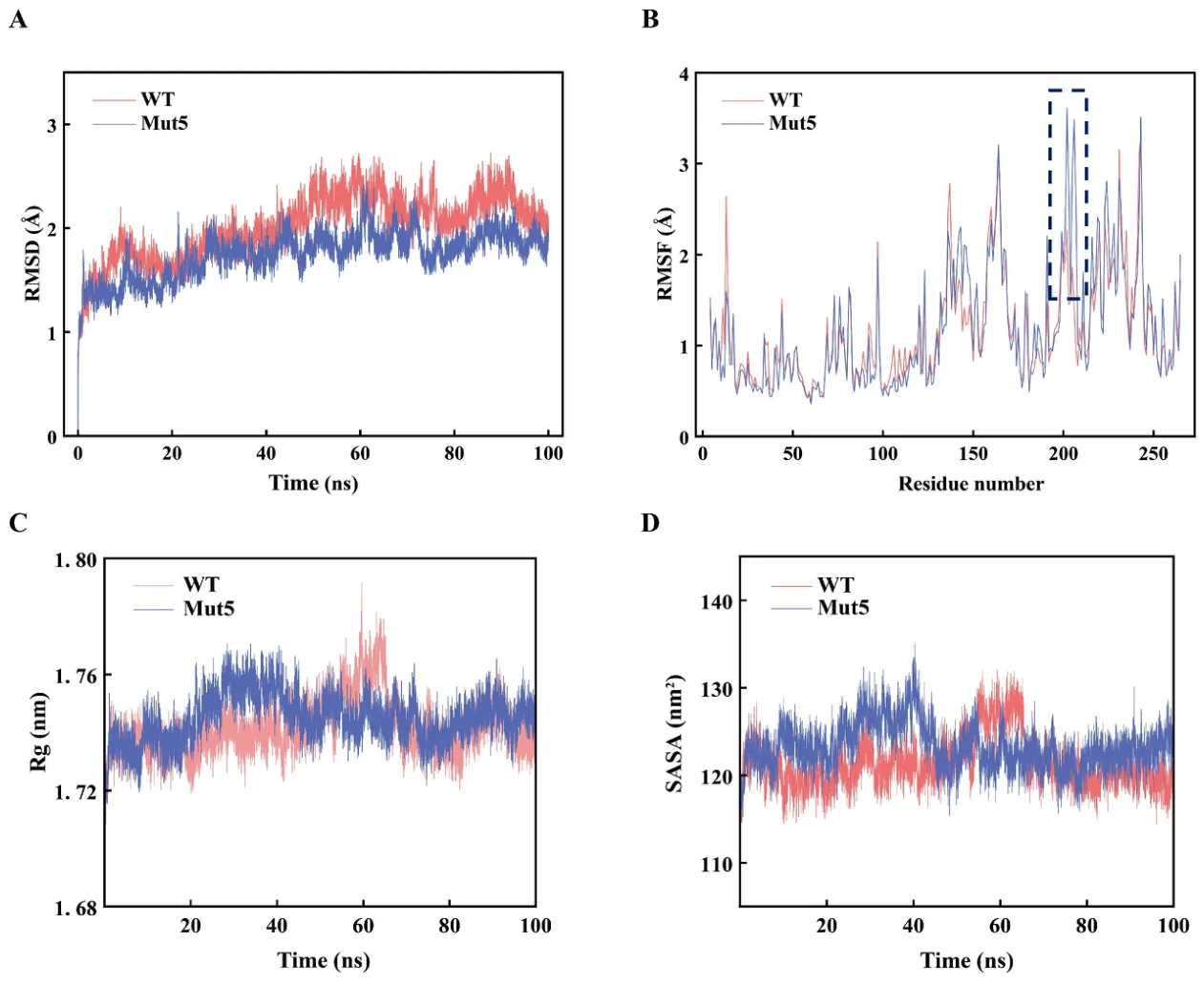
MD simulation analysis of the conformational stability of WT and Mut5. (**A**) RMSD curves; (**B**) RMSF comparison plots. The dotted box indicates areas with relatively obvious differences; (**C**) Rg curves; (**D**) SASA curves.

To investigate the mechanisms underlying changes in enzyme thermostability at the atomic level, 100 ns MD simulations were performed on WT and Mut5. RMSD is a key metric for assessing protein structural stability in molecular dynamics simulations. The results show that the RMSD curves for both complexes remained stable throughout the simulation period. Notably, the average RMSD of Mut5 (0.175 Å) was lower than that of WT (0.201 Å), indicating that its overall structure was more stable (Figure 8A). RMSF is used to assess the degree of fluctuation of individual atoms or residues in proteins or other biomolecules during simulation. The RMSF profiles of WT and Mut5 were broadly similar, with modest localized differences at several residue positions around 200 (Figure 8B, indicated by the dotted box). The radius of gyration (Rg) was used as a measure of overall structural compactness, whereas the solvent-accessible surface area (SASA) was used to assess the extent of protein surface exposure to solvent. The average Rg values for the WT and Mut5 were 1.741 nm and 1.745 nm, respectively (Figure 8C), and the average SASA values were 121.368 nm^2^ and 123.272 nm^2^, respectively (Figure 8D). The values for both were extremely close and tended to stabilize after approximately 40 ns. This indicates that the mutations did not cause significant expansion or collapse of the overall protein structure; both variants maintained stable three-dimensional folding and compactness after binding to the substrate.

### 2.9. Principal Component and Free Energy Landscape Analysis

**Figure 9:**
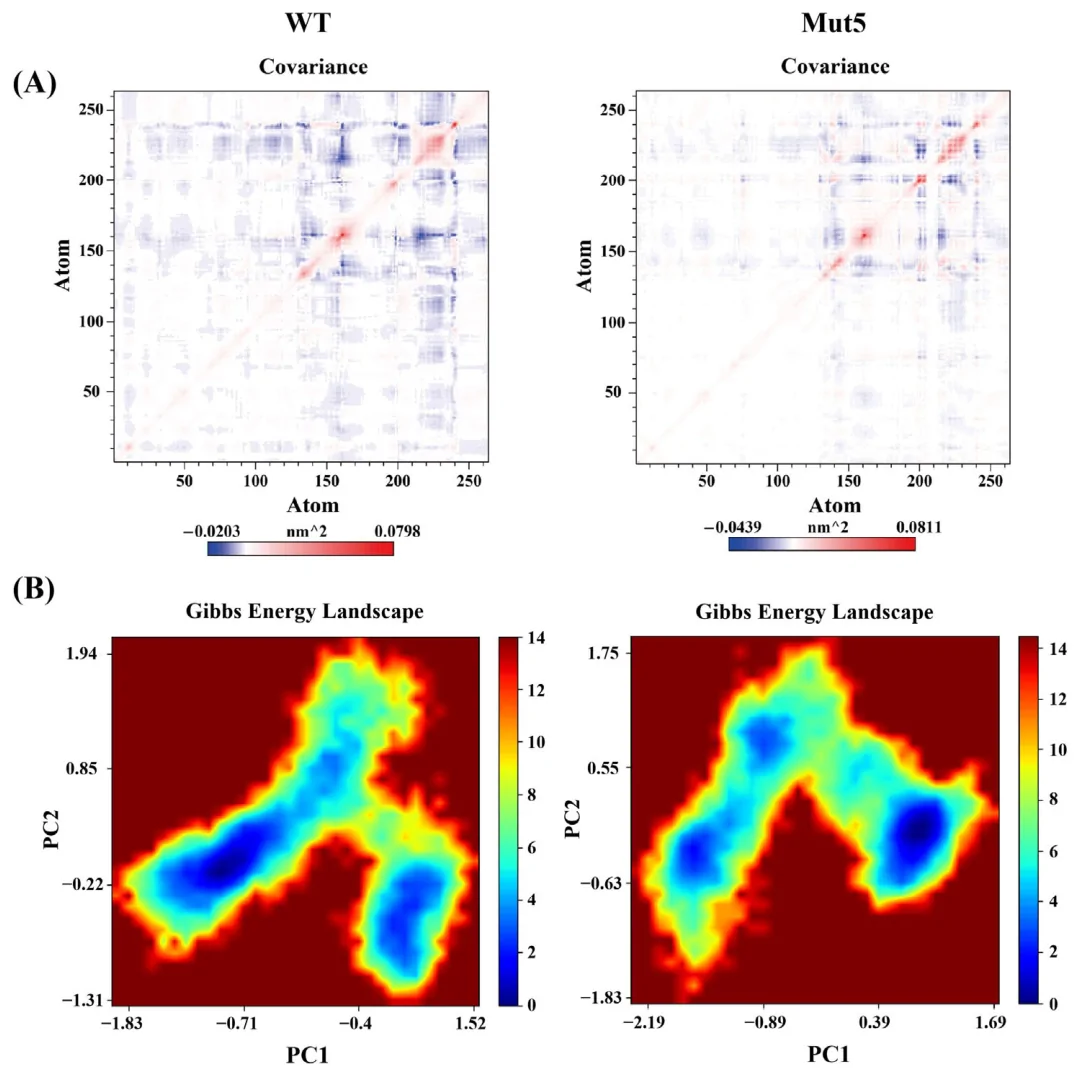
Principal component analysis and free energy landscape characteristics. (**A**) PCA scatter plots and covariance matrices of WT and Mut5; (**B**) FEL of WT and Mut5 on the PC1-PC2 projection plane.

The covariance matrix from principal component analysis (PCA) showed that WT and Mut5 had similar overall numerical ranges (−0.02 to −0.08 nm^2^); however, the mutations caused a reorganization of correlated motion patterns in key regions, indicating that the mutant formed a more coordinated internal dynamical network (Figure 9A). The free energy landscape (FEL) on the PC1-PC2 projection plane demonstrated that the global free energy minimum of Mut5 (−2.19 kcal/mol) was significantly lower than that of the wild type (−1.83 kcal/mol), with a deeper energy basin and a slightly higher energy barrier along the PC1 direction (Figure 9B). This suggests that the dynamic flexibility of the dominant conformation of the mutant was moderately constrained, resulting in higher thermodynamic stability.

## 3. Discussion

Among the enzymatic strategies currently reported for ZEN degradation, laccases and peroxidases often require chemical mediators. These include synthetic compounds such as ABTS, TEMPO, and 1-HBT, as well as naturally occurring mediators such as eugenol and ferulic acid [27,28]. These mediators may themselves exhibit cytotoxicity and are prone to generating oxidative intermediates of unknown structure or with potential toxicity following the reaction [8,29]. In contrast, microbial-derived lactonases of the ZHD family, which function without the requirement of cofactors, are considered a safer alternative [11]. In this study, a novel degrading enzyme, ZHD30L, was identified from *Knufia peltigerae*. Bioinformatics alignment revealed that it shares 71.7% homology with the classical ZHD_101 and retains the typical Ser-Glu-His catalytic triad of hydrolases [30]. This specific spatial configuration is the hallmark core of the classical α/β hydrolase fold, capable of precisely mediating the nucleophilic attack targeting the ester bond of the 14-membered lactone ring [31]. Enzymatic characterization demonstrated that the optimal reaction conditions for recombinant ZHD30L are pH 9.0 and 40 °C. As reported by Wang et al., the optimal temperature and pH for the hydrolase Zhd518 are 40 °C and 8.0, respectively [32]; ZenR exhibits maximum activity at pH 8.0–9.0 and 55 °C [33]; and the optimal conditions for the hydrolase ZHD_LD derived from *Exophiala spinifera* are 50 °C and pH 9.0 [34]. This preference of ZHD30L for weakly alkaline and mesophilic environments is consistent with the catalytic attributes of most reported ZHD family lactonases. However, this enzyme rapidly inactivates at temperatures of 40 °C and above, failing to overcome the prevalent thermostability deficiency inherent in wild-type ZHDs, thereby highlighting the necessity for subsequent molecular modification. Most tested metal ions had little effect on ZHD30L activity under the assay conditions, whereas Cu^2+^, Ni^2+^, and Zn^2+^ caused varying degrees of inhibition. A similar phenomenon was also observed in ZENM [35], ZENG [10], and ZHD-P [36]. From an application perspective, the compatibility of ZHD30L with mineral premixes or metal-rich feed matrices should therefore be evaluated during formulation development. However, the metal-ion concentration used in this initial screening was 5 mM, and the relevance of these effects under actual feed conditions remains to be established.

Determining the structure of the degradation products of the toxin substrate is essential for evaluating the actual detoxification efficacy of the enzyme [30]. Using LC-MS/MS technology, this study showed that ZHD30L hydrolyzes the lactone ring of ZEN to generate the primary product HZEN. This degradation mechanism characterizes ZEN hydrolysis by this class of enzymes. Takahashi-Ando et al. initially identified the ZHD family and confirmed, via nuclear magnetic resonance and other techniques, that the final product is a decarboxylated, non-toxic analog [7]. Subsequent analyses by Liu et al. on the degradation products of recombinant ZHD also reproduced the same intermediate transformation process [30,37]. An intact 14-membered lactone ring is the core structural prerequisite for ZEN to exert its estrogenic toxicity [4]; once this macrocyclic structure is disrupted by enzymatic hydrolysis, the degradation products lose their conformational affinity for the target estrogen receptors [38]. The generation of the non-toxic product HZEN following the enzymatic hydrolysis of ZEN by ZHD30L further supports the high application safety of this enzyme. An additional ion consistent with a product 44 Da lower than HZEN in our study (Figure S2) was also detected. Hu et al. previously reported that the corresponding ring-opened ZEN product underwent partial conversion to a putative decarboxylated product in a control system without *Rhodococcus erythropolis* HQ [33]. They further showed that the stability of this intermediate was solvent-dependent: it was relatively stable in acetonitrile and acetone but degraded in water, methanol, and ethanol. Nevertheless, because the samples in the present study were concentrated and reconstituted in methanol before analysis, and because an acidified aqueous mobile phase and electrospray ionization were used, the stage at which the putative decarboxylation occurred cannot be determined. Formation of DHZEN in the reaction solution therefore remains a proposed interpretation that requires confirmation.

To overcome the limitations of conventional directed evolution, a semi-rational design approach was used, integrating the PROSS algorithm with the Rosetta energy function. By quantitatively evaluating amino acid substitutions through the calculation of ΔΔG changes [23], 50 potential thermostable mutation sites were identified. However, during multi-site stacking, simple combinations of sites often cause negative epistasis due to steric clashes between amino acid residues, which can lead to localized protein rigidification or loss of activity [39]. Therefore, an iterative evolution strategy was employed [40]: using the optimal double mutant E11T-A127V as the scaffold, multiple rounds of site stacking and negative selection were performed under increasing heat-treatment pressure. This process yielded the five-site combinational variant Mut5 (E11T-A127V-I180K-H235P-V156I). During the modification of a lactonase from *Gliocladium roseum*, the screened mutant S162P/S220R exhibited a t_1/2_ at 55 °C that was 36.8 times longer than that of the wild-type, and its *T*_m_ increased significantly by 8.2 °C [14]. When ZENM derived from *Monosporascus* sp. was engineered, the resulting mutant A59P exhibited a t_1/2_ at 55 °C that was 9.8 times longer than that of the wild-type strain, and its *T*_m_ value increased by 7.6 °C [24]. In this study, compared with the wild type, the t_1/2_ of Mut5 at 48 °C was extended from 1.67 min to 796.8 min (an approximately 477-fold increase), the *T*_m_ increased by 10.48 °C, and the *k_cat_*/*K_M_* value improved from 19.08 μM^−1^·s^−1^ (95% Cl = 12.80 to 25.36 μM^−1^·s^−1^) to 33.29 μM^−1^·s^−1^ (95% Cl = 23.20 to 43.38 μM^−1^·s^−1^) (Table 1). The substantial improvement in thermal lifetime suggests that Mut5 is a promising candidate for further application-oriented evaluation. Nevertheless, its performance has so far been demonstrated only in defined laboratory reaction systems, and its stability and catalytic efficiency during actual feed processing, and storage remain to be established.

To explain this, an in-depth exploration was conducted at the molecular structure level. The internal packing density and the conformational entropy of the surface regions of proteins influence the thermodynamic state of enzymes [41]. The microscopic mechanisms underlying the enhanced thermostability of Mut5 can be primarily attributed to the synergistic effects of strengthened electrostatic interactions, increased hydrophobic packing, and main-chain rigidification. First, regarding electrostatic interactions and polar networks, the introduction of a strong positive charge by the I180K mutation reshaped the local charge microenvironment. The salt bridge networks on the protein surface typically serve as the primary structural basis for resisting high-temperature denaturation [42]. Research by Cao et al. confirmed that targeted lysine introduction can induce the formation of a novel salt bridge network, significantly improving the thermal tolerance of the enzyme by strengthening local electrostatic interactions [43]. Second, regarding hydrophobic packing, the A127V and V156I mutations introduced hydrophobic residues with larger side chains to fill internal protein cavities, reinforcing the van der Waals forces of the core backbone. The thermodynamic theory of protein folding indicates that eliminating internal micro-cavities can maximize the hydrophobic collapse effect and expel internal water molecules, thereby contributing substantial stabilization free energy [44]. Zhang et al. also demonstrated that cavity filling can effectively enhance the internal hydrophobic network of the protein, thereby improving overall thermostability [45]. Concurrently, the E11T and H235P mutations reduced local conformational entropy through their distinct chemical properties. Decreasing the conformational degrees of freedom of the unfolded state is the most classical strategy for increasing the *T*_m_ value of a protein [46]. The proline introduced by H235P contains a distinctive pyrrolidine ring structure, which restricts the rotation of main-chain dihedral angles, effectively decreasing the conformational entropy of this local region upon heating [47]. Meanwhile, the threonine introduced by E11T features a β-carbon branch; this branch, situated close to the backbone, increases local steric hindrance, similarly restricting main-chain flexibility [48]. Overall, the five mutation sites enhanced the thermal tolerance of Mut5 through a triple mechanism: establishing salt bridges, filling cavities, and increasing backbone rigidity.

Furthermore, through a 100 ns molecular dynamics simulation, this study clarified the mechanism behind the simultaneous enhancement of thermostability and catalytic efficiency in Mut5 from the perspective of microscopic dynamics. Computational results showed that Mut5 had a significantly lower global minimum in the free energy landscape and a decreased RMSD, confirming its global folding stability against high-temperature thermal perturbations. The modification of ZHD by Guan et al. and the comparative study on thermophilic and mesophilic serine proteases by Sang et al. both cited the reduction in RMSD and the deepening of FEL energy basins as evidence for a substantial increase in enzyme thermostability [25,49]. Although the overall structure tended toward rigidity, the RMSF values of Mut5 in core functional regions showed an upward trend. Essential flexible conformational fluctuations of the enzyme active site are necessary for efficient substrate binding and product release [50]. Studies have shown that the dynamic interplay of synergistic global rigidification and local flexibilization can prevent the limitation that excessive rigidity imposes on catalytic turnover, thereby preserving the flexibility of the substrate channel and maintaining high catalytic activity at elevated temperatures [51]. Additionally remodeling the local polar network shortens the hydrogen bond distance between the substrate and the key residue Thr133. During the binding phase of the enzymatic reaction, the precise desolvation of the substrate and the strengthening of the transition state hydrogen bond network are the core thermodynamic driving forces that lower the catalytic energy barrier [52]. This shortening of the hydrogen bond distance effectively compensates for the energy consumed by polar solvation, resulting in higher substrate affinity. In studies of predictive models for the motional binding paradigm of ZEN hydrolases, Zhi et al. noted that shortening the binding distances of specific residues, increasing hydrophobic packing, and reshaping local interaction networks are crucial for enhancing substrate affinity [53]. This series of structural and thermodynamic reconstructions reveals the underlying molecular logic in the simultaneous improvement in the catalytic activity and thermostability of Mut5.

Several recently engineered ZEN hydrolases have shown substantial improvements in thermal stability (Table 2). The t_1/2_ of ZENM^A59P^ at 55 °C increased 9.8-fold [24], whereas the combined ZENG^S162P/S220R^ variant showed a 36.8-fold increase in t_1/2_ at 55 °C and retained approximately 90% activity after a 10 min treatment at this temperature [14]. Thermostability engineering of ZHRnZ also improved its residual activity at elevated temperatures, and the E122R variant exhibited a 1.3-fold increase in catalytic efficiency [11]. Introducing the T229C/D170C substitutions into ZHD101 increased its t_1/2_ at 50 °C by approximately 200% and increased its apparent *T*_m_ by 18.1 °C [54]. In the present study, Mut5 displayed an approximately 477-fold increase in t_1/2_ at 48 °C and a 10.48 °C increase in apparent *T*_m_ relative to ZHD30L WT. This large fold increase primarily reflects the successful stabilization of a highly thermolabile parental enzyme. Because the reported enzymes were evaluated under different temperatures, buffers, enzyme concentrations, and incubation protocols, these values should be interpreted as within-study improvements rather than as a direct ranking of absolute thermostability.

Several limitations of the present study should be acknowledged. First, the biochemical characterization was performed in defined buffer systems rather than in naturally contaminated feed or food matrices. Components of complex matrices may influence enzyme accessibility, stability, and substrate availability. Second, Mut5 was not evaluated under industrial operations such as feed conditioning, pelleting, extrusion, drying, or long-term storage. Third, the feasibility of large-scale expression, downstream purification, formulation, and cost-effective production was not assessed. Fourth, the detoxification efficacy of Mut5 has not yet been evaluated through estrogen-receptor assays, cell-based models, feeding trials, or in vivo toxicity studies. Finally, the structural interpretations derived from docking and molecular-dynamics simulations remain computational hypotheses that require further experimental validation. These issues will be addressed in future application-oriented studies.

## 4. Conclusions

In this study, a previously uncharacterized ZHD-family zearalenone lactone hydrolase from *Knufia peltigerae* was identified and engineered. Mut5 showed a 10.48 °C increase in apparent *T_m_* and an approximately 477-fold increase in half-life at 48 °C. Its apparent *k_cat_*/*K_M_* was higher than that of WT, whereas the overlapping *K_M_* values indicated broadly comparable substrate-binding characteristics. Computational analyses suggested several possible structural contributions to the improved phenotype, although these interpretations require further experimental validation. Future studies will evaluate the performance of Mut5 in naturally contaminated feed matrices and under industrially relevant operations, including pelleting, storage, and formulation. Large-scale production and formulation studies will also be needed to assess manufacturing feasibility. In addition, estrogen-receptor assays, cell-based toxicity evaluations, and controlled animal-feeding studies will be required to confirm detoxification efficacy and in vivo safety.

## 5. Materials and Methods

### 5.1. Strains, Plasmids, and Reagents

*E. coli* DH5α, BL21(DE3), and pET28a(+) used in this study are preserved in our laboratory. The full gene sequence of ZHD30L was synthesized by GentleGen Co., Ltd. (Suzhou, China). Primer synthesis (Supplementary Table S1) and sequencing identification were provided by Sangon Biotech Co., Ltd. (Shanghai, China). The zearalenone (ZEN) standard was purchased from Pribolab Bioengineering Co., Ltd. (Qingdao, China).

### 5.2. Gene Mining, Phylogenetic, and Sequence Analysis of the Target Enzyme

Using ZHD101 from *Clonostachys rosea* (GenBank: AB003023.1) as a template [7], we identified a target enzyme from *Knufia peltigerae* and named it ZHD30L. Protein sequences were aligned using ESPript 3.0 with default parameters [55]. The phylogenetic tree was constructed in MEGA 7.0 using the Neighbor-Joining method [56]. Evolutionary distances were calculated with the Poisson correction model, gaps were with the complete deletion option, and branch support was evaluated using 1000 bootstrap replicates. All other parameters were kept at their default settings.

### 5.3. Expression and Purification of Recombinant Enzymes

Recombinant strains harboring the target plasmid were inoculated into LB liquid medium supplemented with 80 μg/mL kanamycin and cultured at 37 °C and 200 rpm until the OD_600_ reached 0.6–0.8. IPTG was then added to a final concentration of 0.4 mM to induce protein expression at 16 °C for 12–15 h. Following induction, cells were harvested by centrifugation at 12,000 rpm for 2 min at 4 °C; the supernatant was discarded and the pellet was resuspended in pre-chilled 20 mM PBS. Ultrasonic disruption was then performed in an ice bath using a 3 mm diameter horn, with the power set to 15 percentin pulse mode (3 s on, 3 s off), for a total ultrasonication time of 15 min. The lysate was subsequently centrifuged at 12,000 rpm for 2 min at 4 °C, and the resulting supernatant was collected.

The crude enzyme solution was filtered through a 0.22-μm membrane, and the fermentation supernatant was purified using a protein purifier (BioLogic LP, Shanghai, China) and a Ni-NTA affinity chromatography column. Gradient elution was performed using elution buffer containing different concentrations of imidazole. Fractions from the target elution peaks were collected, and their purity was verified by SDS-PAGE. Following dialysis and desalting, the purified protein was concentrated by ultrafiltration and stored at 4 °C without cryoprotectants prior to analysis; it did not undergo any freeze–thaw cycles. For preliminary screening, total protein concentrations in the clarified mutant lysates were determined using the Bradford assay and adjusted to the same concentration with PBS. Following candidate selection, WT and Mut5 were purified by Ni-NTA affinity chromatography, and equal concentrations of purified enzyme were used for quantitative thermal and kinetic characterization. Unless otherwise stated, biochemical experiments were performed using three independently expressed and purified enzyme preparations. Data are presented as mean ± standard deviation (mean ± SD, n = 3 independent enzyme preparations).

### 5.4. Determination of the Enzymatic Properties of ZHD30L

Most of these ZHD101-like enzymes have an optimal temperature of 30–40 °C and become completely inactive after incubation at 45–50 °C for a few minutes [5]. The optimal reaction temperature was determined within the range of 30–80 °C, with reactions conducted for 3 min at 10 °C intervals. Enzyme solutions were incubated at 40 and 48 °C for various durations (3, 5, 10, 15, 20, 30, 60 min) according to previous research [36], and the residual enzyme activity was measured at the optimal reaction temperature to evaluate thermostability.

The optimal reaction pH was determined at the optimal temperature using 50 mM buffers: NaAc-HAc (pH 3.0–6.0), Na_2_HPO_4_-NaH_2_PO_4_ (pH 6.0–7.0), Tris-HCl (pH 7.0–9.0), and Gly-NaOH (pH 9.0–11.0). The enzyme solution was diluted to an appropriate concentration with different pH buffers and treated at 4 °C for 12 h. pH stability was then measured at the optimal temperature [36].

Under optimal reaction conditions, various metal ions (Ca^2+^, Cu^2+^, Fe^2+^, Fe^3+^, K^+^, Li^+^, Mg^2+^, Mn^2+^, Ni^2+^, and Zn^2+^) were individually added to the reaction system at a final concentration of 5 mM [36]. A reaction system without metal ions served as the control to determine the effects of different metal ions on enzyme activity.

### 5.5. Determination of ZEN Degradation Activity and Identification of Product Structure

Enzyme activity was determined using high-performance liquid chromatography (HPLC). The mutant crude enzyme solutions were diluted to the same concentration with PBS buffer for the initial screening of the mutant library. A 200 μL aliquot of each solution was placed in a 40 °C water bath for heat treatment. Immediately after treatment, the samples were placed on ice to stop thermal denaturation. A 75 μL aliquot of the heat-treated enzyme solution was added to a pre-warmed mixture of substrate (5 μL) and buffer (170 μL). The degradation reaction proceeded under optimal conditions for 3 min, after which 750 μL of ice-cold methanol was added to terminate the reaction, as described previously [36]. The terminated reaction mixture was filtered through a 0.22 μm organic filter membrane, and the residual amount of ZEN in the substrate system was detected by HPLC [10]. The chromatographic conditions were as follows: an Aresil C18 column (4.6 × 250 mm, 5 μm) was used, with a mobile phase of 80% aqueous methanol, a flow rate of 1 mL/min, a column temperature of 30 °C, and a detection wavelength of 245 nm.

To identify the structure of the ZEN degradation product, the purified enzyme was used and the reaction solution was concentrated by rotary evaporation and reconstituted in chromatographic-grade methanol, followed by detection using high-resolution quadrupole time-of-flight liquid chromatography–tandem mass spectrometry (LC-MS/MS) (Xevo G2-XS QTof, Waters Corporation). Chromatographic separation was performed using a C18 column (2.1 × 100 mm, 1.7 μm), with a mobile phase consisting of an aqueous formic acid solution (phase A) and chromatographic-grade acetonitrile (phase B). Mass spectrometry data were acquired in negative ion mode (ESI^−^). The structure of the ZEN hydrolysis product was determined by extracting the exact mass-to-charge ratio (*m*/*z*) of the precursor ion and the secondary fragment ions of the degradation product [57].

### 5.6. Homology Modeling and Mutant Construction

The three-dimensional (3D) structure of the ZHD30L was constructed using the SWISS-MODEL server [58]. PROSS server was used to predict 50 potential thermostable mutation sites combined with sequence conservation analysis with Rosetta folding free energy (ΔΔG) calculations [20,59]. Mutants were constructed using the whole-plasmid PCR method [60]. The recombinant plasmid pET28a-ZHD30L was used as a template for amplification with specific primers. PCR products were digested with DpnI to remove the methylated template DNA and then transformed into *E. coli* DH5α competent cells. After verification by sequencing, the constructs were transformed into the expression host *E. coli* BL21 (DE3) to generate a single-point mutant library. Multi-point combined mutants were constructed using an iterative superposition strategy [61]: the screened single-point mutants with enhanced thermostability were first used as initial scaffolds to build double-combination mutants. For each subsequent round of constructing higher-order combinations, the best-performing mutant from the current round served as the new scaffold for stacking the remaining beneficial sites. Throughout the iterative process, negative-effect sites that caused a decline in stability were systematically eliminated.

### 5.7. Detection of Secondary Structure, Melting Temperature, Half-Time and Kinetic Parameters

Secondary structure and melting temperature (*T*_m_): The secondary structures and *T*_m_ values of WT and Mut5 were determined using a circular dichroism (CD) spectropolarimeter. Protein samples were diluted to 0.3 mg/mL, and full-wavelength scans in the far-UV spectrum were performed within the wavelength range of 190–260 nm. The change in ellipticity was monitored at 220 nm, with the heating range set from 10–80 °C at a heating rate of 1 °C/min, to calculate the *T*_m_ value of the proteins [62].

Half-life (t_1/2_): The purified WT and the optimal combined mutants from each round were incubated in a 48 °C water bath for different time intervals [14,63]. The residual enzyme activity was measured, and the t_1/2_ was calculated according to the method described by Fang et al. [14].

Under optimal reaction conditions, using different concentrations of ZEN (0.0025, 0.005, 0.01, 0.02, 0.04, and 0.08 mg/mL) as the substrate. The experiments were performed in triplicate using independently prepared reaction mixtures to determine the kinetic parameters for WT and Mut5. The kinetic parameters were obtained by nonlinear fitting to the Michaelis-Menten equation and are reported as mean ± SD (n = 3).

### 5.8. Molecular Docking and MD Simulations

Molecular docking of the ligand ZEN was performed using AutoDock Vina 1.2.0 based on the Lamarckian genetic algorithm [64]. The docking grid was set to encompass the active site of the target protein with dimensions of 20 × 20 × 20 Å^3^, and the exhaustiveness parameter was set to 50. The binding conformation with the highest score was selected as the optimal complex structure for subsequent MD simulations.

After selecting the optimal binding conformation, MD simulations were conducted using GROMACS 2023.05 software and the CHARMM36 force field [65,66]. The complex was solvated in a TIP3P water box, and 150 mM NaCl was added to neutralize the system charge. Following energy minimization and equilibration under NVT (310 K) and NPT (1 atm) ensembles, a 100 ns production simulation was performed. The MM/GBSA method implemented in AmberTools25 was used to extract the equilibrated 20 ns trajectory for calculating the binding free energy [26]. Additionally, metrics including root mean square deviation (RMSD) and root mean square fluctuation (RMSF) were extracted to analyze the conformational dynamic characteristics.

## Figures and Tables

**Table 1:** Comparison of enzyme kinetic parameters between WT and Mut5.

**Enzyme**	***K_M_*** **[95% Cl] (μM)**	***k_cat_*** **[95% Cl] (s** **^−1^** **)**	***k_cat_*** **/*K_M_*** **[95% Cl] (** **μ** **M** **^−1^** **·** **s** **^−1^** **)**
WT	74.77 [57.17 to 92.37]	1427 [1100.52 to 1753.48]	19.08 [12.80 to 25.36]
Mut5	64.41 [49.99 to 78.83]	2144 [1705.16 to 2582.84]	33.29 [23.20 to 43.38]

**Table 2:** Comparison of the thermostability characteristics of representative engineered ZEN hydrolases.

Engineered Enzyme	Source Organism	Optimal Reaction Conditions	Reported Thermostability Improvement	Reference
ZENM^A59P^	*Monosporascus* sp. GIB2	55 °C, pH 9.0	At 55 °C, half-life (t_1/2_) increased from 2.85 to 27.84 min (9.8-fold); *T*_50_ increased by 7.6 °C; apparent *T*_m_ increased by 23.20 °C.	[24]
ZENG^S162P/S220R^	*Gliocladium roseum*	38 °C, pH 7.0	At 55 °C, t_1/2_ increased by 36.8-fold; apparent *T*_m_ increased by 8.2 °C; approximately 90% residual activity after 10 min at 55 °C.	[14]
ZHRnZ^E122Q/E122R^	*Rosellinia necatrix*	45 °C, pH 9.0	>30% residual activity after 2 min at 50 °C; approximately 10% after 1 min at 60 °C; E122R showed a 1.3-fold increase in catalytic efficiency	[11]
ZHD101^T229C/D170C^	*Clonostachys rosea*	45 °C, pH 9.0	At 50 °C, t_1/2_ increased by approximately 200%; *T*_50_ increased by 7 °C; apparent *T*_m_ increased by 18.1 °C	[54]
ZHD30L^E11T/A127V/I180K/H235P/V156I^	*Knufia peltigerae*	40 °C, pH 9.0	At 48 °C, t_1/2_ increased from 1.67 to 796.8 min (approximately 477-fold); apparent *T*_m_ increased by 10.48 °C	This study

## Data Availability

The original contributions presented in this study are included in the article/Supplementary Material. Further inquiries can be directed to the corresponding author.

## Supplementary Materials

The following supporting information can be downloaded at https://www.mdpi.com/article/10.3390/toxins18080324/s1: Figure S1: Enzymatic properties of ZHD30L. (A) pH stability of ZHD30L; (B) Effects of metal ions on the enzyme activity of ZHD30L; Figure S2. (A) MS/MS spectra of HZEN and DHZEN; (B) The process by which hydrolases degrade ZEN; Table S1: Primers used in this study.
