# Supplementary material for: Identification and Thermal Stability Modification of a Zearalenone Lactone Hydrolase ZHD30L
Source: Toxins (Basel). 2026 Jul 26;18(8):324. doi: 10.3390/toxins18080324 (PMC13517648; doi:10.3390/toxins18080324)
Supplement: Supplementary file 1 [file toxins-18-00324-s001.zip › toxins-4433237-supplementary.pdf]

Supplementary Figures

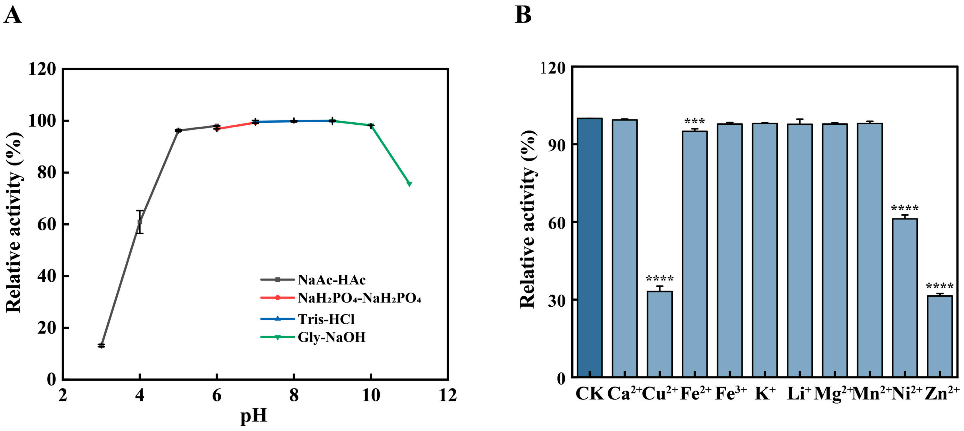

Figure S1. Enzymatic properties of ZHD30L. (**A**) pH stability of ZHD30L; (**B**) Effects of metal ions on the enzyme activity of ZHD30L.

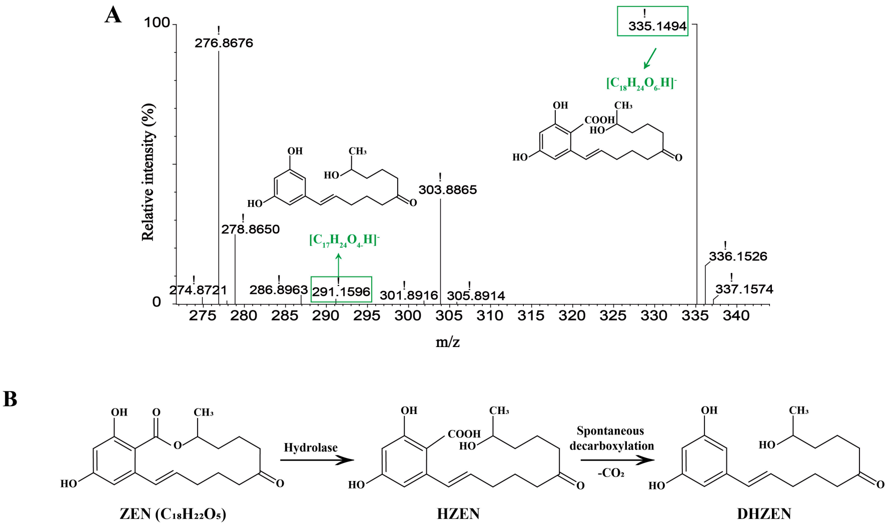

Figure S2. (A) MS/MS spectra of HZEN and DHZEN; (B) The process by which hydrolases degrade ZEN.

Supplementary Table

Table S1. Primers used in this study

Primer Name	Primer sequences (5′-3′)
S3P-F	ccatATGGTGCCAGCCCGTATTCGCAGCACC
S3P-R	GAATACGGGCTGGCACCATatggctgccgcgcg
T10I-F	TCGCAGCACCATCGAAACCAAAGATGGCATTAAATGG
T10I-R	CTTTGGTTTCGATGGTGCTGCGAATACGGGCGC
K76Q-F	GGAAACCTATCAGGATGTGACCGGCCATAAACTG

K76Q-R	CGGTCACATCCTGATAGGTTTCCGGCGGGGCTTC
E92D-F	GATACCCTGCTGGATCACCTGGGCATTAAAGAC
E92D-R	TGCCCAGGTGATCCAGCAGGGTATCCACGTAGG
S99A-F	CATTAAAGACGCATCGGTCTGGGGCTGCTCTAG
S99A-R	CCCAGACCGATGCGTCTTTAATGCCCAGGTG
C115V-F	GCTGGCGCTGGTGGCGGCATTCCCGGAACGTGT
C115V-R	GGAATGCCGCCACCAGCGCCAGCACGGTGGTGG
A117F-F	GCTGTGTGCGTTCTTCCCGGAACGTGTGCGCAATG
A117F-R	GTTCCGGGAAGAACGCACACAGCGCCAGCACGG
A127P-F	CAATGCCATGCCCCATGAACTGCCGATGACC
A127P-R	GCAGTTCATGGGGCATGGCATTGCGCACACGTTC
N134L-F	GCCGATGACCCTCCCGGAAAAACTGCACGGAAT
N134L-R	GTTTTTCCGGGAGGGTCATCGGCAGTTCATG
N142Y-F	GCACGGAATTTACGATCTGGACCCGGCCGAAATTG
N142Y-R	GGTCCAGATCGTAAATTCCGTGCAGTTTTTCCG
V156F-F	AATGGCGGCATTCACCCGTTTTTTTAGCGGCGG
V156F-R	GCTAAAAAAACGGGTGAATGCCGCCATTTTATCGGCAATTTC
I180K-F	CCGCCTGCACAAAAACTATCCGCGTTGGACCGC
I180K-R	GCGGATAGTTTTTGTGCAGGCGGGCATGCACTTC
T186A-F	TCCGCGTTGGGCCGCGGGCTACCCGCGCACCAT
T186A-R	GGTAGCCCGCGGCCCAACGCGGATAGTT
A187R-F	GCGTTGGACCCGGGGCTACCCGCGCACCATTCC
A187R-R	GCGGGTAGCCCCGGGTCCAACGCGGATAGTT
S216Y-F	CGTGGGCGCGTACACCCCGAGCGAAGCCTTCTG
S216Y-R	CGCTCGGGGTGTACGCGCCCACGGTCCAATCAATC
S219L-F	GAGCACCCCGCTCGAAGCCTTCTGGGAAAACTT
S219L-R	CAGAAGGCTTCGAGCGGGGTGCTCGCGCCCACGG

A221W-F	CCCGAGCGAATGGTTCTGGGAAAACTTTGTGATC
A221W-R	TTTCCCAGAACCATTCGCTCGGGGTGCTCGCGC
W223F-F	CGAAGCCTTCTTCGAAAACTTTGTGATCGCCGG
W223F-R	CAAAGTTTTCGAAGAAGGCTTCGCTCGGGGTGC
V227M-F	GGAAAACTTTATGATCGCCGGCCGCGAAGGTATTC
V227M-R	GGCCGGCGATCATAAAGTTTTCCCAGAAGGCTTC
G230W-F	TGTGATCGCCTGGCGCGAAGGTATTCATATTGG
G230W-R	TACCTTCGCGCCAGGCGATCACAAAGTTTTCCC
R231K-F	GATCGCCGGCAAGGAAGGTATTCATATTGGCAT
R231K-R	GAATACCTTCCTTGCCGGCGATCACAAAGTTTTC
T260I-F	TGTGGTCGATACCTGCCGTAAATATCTGTAAgc
T260I-R	GATATTTACGGCAGGTATCGACCACATATTTCGCAAAC
A4E-F	tATGGTGAGCGAACGTATTCGCAGCACCACCGA
A4E-R	TGCGAATACGTTCGCTCACCATatggctgccgc
E11T-F	CAGCACCACCACAACCAAAGATGGCATTAAATG
E11T-R	CATCTTTGGTTGTGGTGGTGCTGCGAATACGGG
V32I-F	TATTGTGCTGATACCGGATGGCCTGGGCGAATG
V32I-R	GGCCATCCGGTATCAGCACAATATCCGGGCCGC
P45A-F	GTTCGACAAAGCGATGAGTATTATTGCCAGCAA
P45A-R	TAATACTCATCGCTTTGTCGAACATCTGGCATT
D88I-F	GACCTACGTGATTACCCTGCTGGAACACCTGGG
D88I-R	CCAGCAGGGTAATCACGTAGGTCGCCAGTTTAT
V101F-F	AGACAGCTCGTTCTGGGGCTGCTCTAGCGGGGC
V101F-R	AGCAGCCCCAGAACGAGCTGTCTTTAATGCCCA
T109A-F	TAGCGGGGCCGCCACCGTGCTGGCGCTGTGTGC
T109A-R	CCAGCACGGTGGCGGCCCCGCTAGAGCAGCCCC
F118Y-F	GTGTGCGGCATACCCGGAACGTGTGCGCAATGC

F118Y-R	CACGTTCCGGGTATGCCGCACACAGCGCCAGCA
A127V-F	CAATGCCATGGTCCATGAACTGCCGATGACCAA
A127V-R	GCAGTTCATGGACCATGGCATTGCGCACACGTT
L130V-F	GGCCCATGAAGTGCCGATGACCAACCCGGAAAA
L130V-R	TGGTCATCGGCACTTCATGGGCCATGGCATTGC
M132T-F	TGAACTGCCGACGACCAACCCGGAAAAACTGCA
M132T-R	CCGGGTTGGTCGTCGGCAGTTCATGGGCCATGG
T133Y-F	ACTGCCGATGTACAACCCGGAAAAACTGCACGG
T133Y-R	TTTCCGGGTTGTACATCGGCAGTTCATGGGCCA
K137D-F	CAACCCGGAAGATCTGCACGGAATTAACGATCT
K137D-R	TTCCGTGCAGATCTTCCGGGTTGGTCATCGGCA
H139K-F	GGAAAAACTGAAAGGAATTAACGATCTGGACCC
H139K-R	CGTTAATTCCTTTCAGTTTTTCCGGGTTGGTCA
H139A-F	GGAAAAACTGGCCGGAATTAACGATCTGGACCC
H139A-R	CGTTAATTCCGGCCAGTTTTTCCGGGTTGGTCA
G140N-F	AAAACTGCACAATATTAACGATCTGGACCCGGC
G140N-R	GATCGTTAATATTGTGCAGTTTTTCCGGGTTGG
I141L-F	ACTGCACGGACTTAACGATCTGGACCCGGCCGA
I141L-R	CCAGATCGTTAAGTCCGTGCAGTTTTTCCGGGT
N142H-F	GCACGGAATTCACGATCTGGACCCGGCCGAAAT
N142H-R	GGTCCAGATCGTGAATTCCGTGCAGTTTTTCCG
A147E-F	TCTGGACCCGGAAGAAATTGCCGATAAAATGGC
A147E-R	CGGCAATTTCTTCCGGGTCCAGATCGTTAATTC
D151K-F	CGAAATTGCCAAGAAAATGGCGGCAGTGACCCG
D151K-R	CCGCCATTTTCTTGGCAATTTCGGCCGGGTCCAG
V156I-F	AATGGCGGCAATAACCCGTTTTTTAGCGGCGG
V156I-R	AAAAACGGGTTATTGCCGCCATTTTATCGGCAA

P183V-F	CATTAACTATGTGCGTTGGACCGCGGGCTACCC
P183V-R	GCGGTCCAACGCACATAGTTAATGTGCAGGCGG
P190I-F	CGCGGGCTACATTCGCACCATTCCGCCGAGCGC
P190I-R	GAATGGTGCGAATGTAGCCCGCGGTCCAACGCG
P195Q-F	CACCATTCCGCAGAGCGCCCCGACCGCGAGCGA
P195Q-R	TCGGGGCGCTCTGCGGAATGGTGCGCGGGTAGC
A200D-F	CGCCCCGACCGACAGCGAAGACCTGCATAAACG
A200D-R	GGTCTTCGCTGTCGGTCGGGGCGCTCGGCGGAA
S219T-F	GAGCACCCCGACCGAAGCCTTCTGGGAAAACTTT
S219T-R	AGAAGGCTTCGGTCGGGGTGCTCGCGCCCACGG
F226V-F	CTGGGAAAACGTTGTGATCGCCGGCCGCGAAGG
F226V-R	CGGCGATCACAACGTTTTCCCAGAAGGCTTCGC
G230T-F	TGTGATCGCCACCCGCGAAGGTATTCATATTGG
G230T-R	TACCTTCGCGGGTGGCGATCACAAAGTTTTCCC
H235P-F	CGAAGGTATTCCTATTGGCATTCTGCCGGGCAG
H235P-R	GAATGCCAATAGGAATACCTTCGCGGCCGGCGA
